# The Use of New Mobile and Gaming Technologies for the Assessment and Rehabilitation of People with Ataxia: a Systematic Review and Meta-analysis

**DOI:** 10.1007/s12311-020-01210-x

**Published:** 2020-11-14

**Authors:** Eleonora Lacorte, Guido Bellomo, Sara Nuovo, Massimo Corbo, Nicola Vanacore, Paola Piscopo

**Affiliations:** 1 National Centre for Disease Prevention and Health Promotion, Italian National Institute of Health, Rome, Italy; 2 Department of Human Neuroscience, Sapienza University of Rome, Rome, Italy; 3 Department of Neurorehabilitation Sciences, Casa Cura Policlinico (CCP), Milan, Italy; 4 Department of Neuroscience, Italian National Institute of Health, Viale Regina Elena, 00161 Rome, Italy

**Keywords:** Video games, Mobile applications, Technology, Rehabilitation, Ataxia, Systematic review

## Abstract

There are no currently available disease-modifying pharmacological treatments for most of the chronic hereditary ataxias; thus, effective rehabilitative strategies are crucial to help improve symptoms and therefore the quality of life. We propose to gather all available evidence on the use of video games, exergames, and apps for tablet and smartphone for the rehabilitation, diagnosis, and assessment of people with ataxias. Relevant literature published up to June 8, 2020, was retrieved searching the databases PubMed, ISI Web of Science, and the Cochrane Database. Data were extracted using a standardized form, and their methodological quality was assessed using RoB and QUADAS-2. Six studies of 434 retrieved articles met the predefined inclusion/exclusion criteria. Two of them were diagnostic, while 4 were experimental studies. Studies included participants ranging from 9 to 28 in trials and 70 to 248 in diagnostic studies. Although we found a small number of trials and of low methodological quality, all of them reported an improvement of motor outcomes and quality of life as measured by specific scales, including the SARA, BBS, DHI, and SF-36 scores. The main reason for such low quality in trials was that most of them were small and uncontrolled, thus non-randomized and unblinded. As video games, exergames, serious games, and apps were proven to be safe, feasible, and at least as effective as traditional rehabilitation, further and more high-quality studies should be carried out on the use of these promising technologies in people with different types of ataxia.

## Background

Ataxia, or lack of voluntary coordination of muscle movements, has an overall prevalence of around 26 per 100,000 in European pediatric population [1], 8.4 per 100,000 (95% CI 7.2 to 11.6) for idiopathic late-onset cerebellar ataxia (LOCA), and 1.8 per 100,000 (95% CI 0.8 to 2.7) for inherited LOCA [2]. Based on the population living in Europe, we can estimate about 525,000 cases in all ages (1300 in pediatric population and 431,200 and 92,500 for idiopathic and inherited LOCA, respectively) (https://ec.europa.eu/eurostat). Ataxia is a common neurological sign that might be due to several different neurological conditions, including brain tumors, brain injuries, stroke, infections (e.g., varicella), toxicity, or genetic causes. Its evolution can be acute, subacute, episodic, or chronic, with the latter including both progressive and non-progressive forms. The diagnosis of the underlying cause is a crucial step, and might be a long process in case of chronic or episodic ataxias, due to the rarity and complexity of these conditions [3]. Cerebellar ataxia is typically among the core features of these diseases, affecting motor skills, eye movements, balance, and coordination, thus significantly affecting activities of daily living (ADL). In some hereditary conditions, ataxia is part of a multisystem dysfunction, which may cause intellectual disability and/or other neurological symptoms, spine deformities such as scoliosis, and/or several other disorders affecting different organs (e.g., cardiomyopathy, congenital cataracts, optic nerve atrophy, retinal dystrophy, and deafness) [4–8].

There are no currently available disease-modifying pharmacological treatments for most of the chronic hereditary ataxias, thus effective rehabilitative strategies are crucial to improve symptoms and therefore the quality of life [9].

Rehabilitative and physiotherapeutic interventions are increasingly being tested in clinical studies for the treatment of people with different ataxias. Previous studies showed that treatments based on demanding gait and balance tasks can increase postural stability and lead to reduce the dependency of patients on walking aids, thus increasing their independence in ADL [10–13].

Non-ambulant patients with more severe ataxias were also shown to benefit from locomotion and treadmill training with or without body weight support [9].

In this framework, some recent studies have shown that the use of some virtual reality tools can be effective for the treatment of people with progressive ataxias [14]. Virtual reality is often incorporated within exergames, which are video games designed as easier, more entertaining, and more appealing exercise tools compared to usual, traditional, training systems. Exergaming is defined as technology-driven physical activities, such as video game play, that requires participants to be physically active or exercise in order to play the game [15]. Exergames usually include coordination and balance tasks and are specifically structured to enhance the participation and adherence of the player/participant to the game/intervention. As rehabilitation for people with ataxia can be challenging, these games could be an extremely powerful and useful tool to improve rehabilitative interventions in these patients [16]. As exergames are usually extremely enjoyable and easy to play, do not require the constant presence of a trainer to monitor the activity, and are usually easily available and not very expensive, including these type of games in the rehabilitation program could also help moving the whole intervention, or part of it, from a clinical setting to a home setting, and could help to turn a rehabilitation session into more enjoyable activities, particularly in children. Moreover, the use of exergames could help to reach a higher number of patients by minimizing the access to the health system, as patients could be able to follow the whole rehabilitation program, or even part of it, at home.

Exergames have been proven as effective in improving motor and cognitive symptoms in several neurological and neurodegenerative diseases [17, 18]. A relatively recent systematic review [19] concluded that consistent evidence supports the effectiveness of rehabilitation in improving motor function, ataxic symptoms, and balance in patients with chronic ataxias, including rehabilitation using serious games and exergames. Furthermore, the therapeutic use of commercially available exergame systems and applications for smartphones and tablets can be very cost-effective and improve quality of life and social inclusion, minimizing the perception of disability. However, no systematic reviews nor meta-analyses are currently available on the effectiveness of this kind of technology specifically in patients with chronic ataxias. For all these reasons, we deemed it relevant to review all existing evidence on the use of this technology in patients with ataxias. Therefore, the objective of this review was to gather, qualitatively assess, and summarize the results, both narratively and through a meta-analysis, from all available evidence on the use of video games, exergames, and apps for tablet and smartphone for the rehabilitation, diagnosis, and assessment of people with ataxias.

## Methods

This systematic review was carried out according to the methodology reported in the Cochrane Handbook for Systematic Reviews [20] and followed the PRISMA statement for reporting systematic reviews and meta-analyses [21, 22]. A structured bibliographic search was performed on the databases PubMed, ISI Web of Science, and the Cochrane Database of Systematic Reviews using the following search terms: (“app” OR “apps” OR “smartphone” OR “smartphones” OR “smart-phone” OR “smart-phones” OR “smart phone” OR “smart phones” OR “tablet*” OR “mobile” OR “game” OR “kinect” OR “nintendo” OR “games” OR “gaming” OR exergam* OR “virtual reality” OR “augmented reality”) AND (*ataxi* OR “joubert*”). No limitations were applied for date of publication, study design, nor language. The bibliographic references of selected studies were also browsed to identify further possibly relevant literature. Two independent reviewers (EL, PP) initially selected studies based on their pertinence with and relevance to the topic of the review. Disagreements were solved by discussion or by a third independent reviewer (NV). Relevant literature published up to June 8, 2020, was retrieved searching the databases. Selected studies were retrieved in full text, and the following predefined inclusion/exclusion criteria were applied. We only included (1) experimental and/or observational studies; (2) studies that reported data on the use of technologies such as computer games, gaming consoles, tablet or smartphone apps, and/or devices for augmented or virtual reality for the rehabilitation and/or treatment of people with ataxia; (3) studies that enrolled people with ataxia of any age class; (4) studies that enrolled patients with any type of ataxia; and (5) studies that reported enough information and data to allow for an adequate quality assessment and a summary of evidence. We excluded (1) conference proceedings, letters, abstracts, editorials, narrative reviews, systematic reviews, meta-analyses, case-reports, or case-series; and (2) studies reporting only narrative or non-quantifiable results. Systematic reviews were not included, but were anyway selected and analyzed to search their bibliographies, and to check for consistency of results.

The methodological quality of all included studies was assessed by 3 independent reviewers (PP, EL, GB) using The Cochrane Collaboration’s tool for assessing risk of bias (RoB)in randomized trials [23], and the QUADAS-2 tool for diagnostic studies [24]. The RoB tool includes seven specific domains aimed at assessing selection bias, performance bias, detection bias, attrition bias, reporting bias, and other potential sources of bias, and requires that enough details are provided to adequately assess the risk of bias, which is defined as “low risk,” “high risk,” or “unclear risk.” The QUADAS-2 tool includes 4 domains analyzing the adequateness of the methodology adopted for patient selection, the choice and management of the index test and the reference standard, and the flow and timing with which the tests were administered. It includes also an assessment of concerns about applicability for 3 of the considered domains (i.e., patient selection, index test, reference standard). The tool does not allow for a global scoring, but it provides an overall rating of high, unclear, or low risk of bias for each domain, and an overall rating of high, unclear, or low concern for applicability for the 3 considered domains. Further potential bias or methodological flaws were also addressed. As neither of the tools provides a method to calculate an overall quality score, we calculated it for both scales. For the RoB tool, the overall score was calculated by summing the number of items scored as “low risk of bias,” thus having an overall score ranging from 0 to 7, with higher scores indicating higher quality. For the QUADAS-2 tool, the score was calculated by summing the number of items scored as “low risk of bias” or “low applicability concern,” thus obtaining an overall score ranging from 0 to 7, with higher scores indicating higher quality.

Data extraction was performed by 3 independent reviewers (GB, PP, EL), and data were summarized in specifically designed standardized forms. Disagreements were resolved by discussion between the reviewers.

Due to a high heterogeneity, a meta-analysis was carried out using data from the only 2 experimental studies reporting results for the same outcome measure. The summary tables for the qualitative assessment and the meta-analysis were performed using the software RevMan version 5.3 provided by the Cochrane Collaboration.

## Results

A total of 661 records were retrieved through the bibliographic searches. No articles were retrieved by browsing the references of included studies. No systematic reviews nor meta-analyses were available on this topic. Of the studies retrieved through bibliographic searches, 7 studies were selected based on their relevance and pertinence to the topic of the review. Full texts were gathered and, after applying the predefined inclusion and exclusion criteria, only 6 studies were included in the review. One study was excluded, as it was a letter to the editors reporting preliminary data on 1 patient who was subsequently enrolled in the study published in 2017 by Schatton et al. [25].

**Fig. 1:**
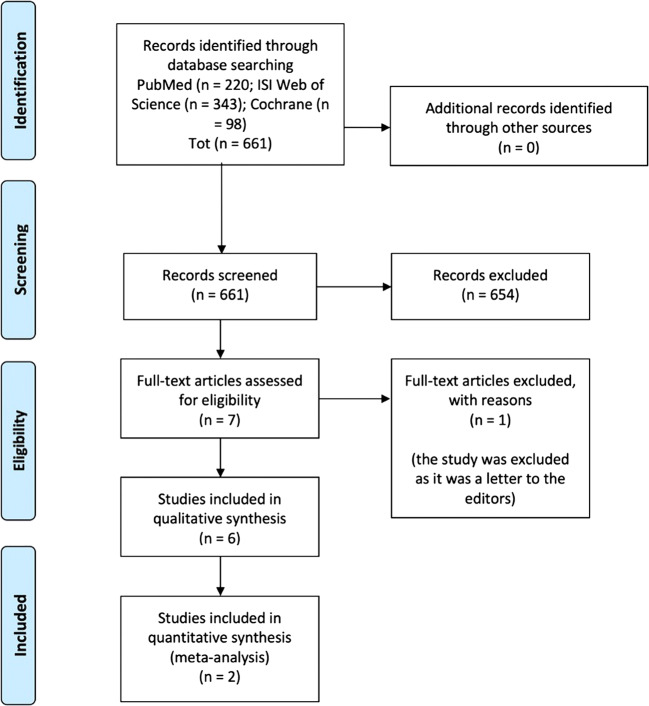
Flow diagram of the literature

The flow diagram of literature is reported in Fig. 1.

### Qualitative Assessment of Included Studies

Four studies were experimental studies, with 1 controlled trial [26] and 3 single-group trials [27–29]. Two studies were categorized as diagnostic studies [30, 31].

All included experimental studies investigated the effectiveness of either available video games for both Nintendo Wii and Microsoft Xbox Kinect [27–29] or specifically developed exergames adopting the Kinect sensor in both children and adults with different ataxias [26].

The 2 diagnostic studies evaluated the accuracy of either an already available serious game coupled with the Kinect sensor [30] or a specifically developed app for tablet and smartphone [31] to assess motor symptoms in patients with ataxias, and to discriminate between healthy subjects and patients with ataxias.

A summary of the results, main characteristics, and quality of included studies is reported in Table 1.

**Table 1:** Summary of the characteristics and results of included studies

Characteristics				Quality assessment*	Summary of findings		
					Number of subjects		Results
First author, year	Definition of participants	Intervention	Attrition	Overall appraisal*	Cases and characteristics	Reference population and characteristics	
Intervention							
Wang, 2018	Adults with genetically confirmed SCA-3 recruited in a Taipei medical center	Intervention: 30-min exergaming with Kinect sensor Control: 30-min conventional balance and coordination training session Treatment length: 4 weeks	14 subjects assessed for eligibility 7 excluded (did not meet inclusion criteria, refused participation) 9 subjects randomized (block randomization) None of the enrolled patients was lost to follow-up.	4	Intervention: 5 Median age 54.0 years (range 51.0–60.0) M 2; F 3 Median disease duration: 6.0 years (range 1.0–16.0)	Control: 4 Median age: 57.0 (range 44.0–61.0) M 2; F 2 Median disease duration: 5.5 years (range 1.0–8.0)	SARA score (total) Median % change from baseline (range) Intervention: − 30.0 (− 42.9–− 25.0) Control: − 16.8 (− 33.3–− 8.3) Directional control of the limit of stability test Median % change from baseline (range) Intervention: forward 7.2 (− 15.6–14.3), more affected side 3.4 (− 10.6, 12.8), less affected side − 8.1 (− 26.6–6.4), backward − 15.5 (− 20.8–− 12.7) Control: forward 3.6 (− 21.4–41.4), more affected side − 5.8 (− 33.7–25.8), less affected side − 7.1 (− 30.2–20.8), backward 63.9 (− 100.0–520.0) Nine-hole peg test Median % change from baseline (range) Intervention: more affected side − 11.7 (− 16.6–2.6), less affected side − 5.1 (− 15.2–4.2) Control: more affected side − 2.0 (− 15.1–0.2), less affected side − 9.8 (− 27.7–9.8) Gait performance Median % change from baseline (range) Intervention: walking speed cm/s − 1.3 (− 1.6–− 0.6), step width cm 9.3 (− 39.3–33.1) Control: walking speed cm/s 3.1 (− 5.8–21.6), step width cm − 0.6 (− 8.6–21.0)
Schatton, 2017	Patients with advanced spinocerebellar disease recruited from the ataxia clinic of the University of Tübingen, Germany	12 weeks (2 consecutive phases of 6-week training at home) of coordinative training specific for trunk training and postural control, based on commercial video games (Nintendo Wii and XBox Kinect)	14 patients were screened for inclusion, but 4 were not included as they were unable to sit freely and 2 had severe mental deficits None of the enrolled patients were lost to follow-up Outcomes measured 2 weeks pre-intervention (E1), pre-intervention (E2), post-phase 1 (E3), and post-phase 2 (E4)	3	11 (M 8; F 3) Mean age 16 years (range 6–29) 2 autosomal-recessive ataxia, 5 Friedreich ataxia, 3 ataxia telangiectasia, 1 ataxia with oculomotor ataxia type 1	NA	SARA score (total) Mean baseline: 20.9 ± 5.8 Change between the 4 assessments: *χ*^2^ = 13.7, *p* = 0.003) E1 to E2: unchanged Pre-post treatment change: average drop of 2.5 points E2 to E3: *p* < 0.002; E2 to E4: *p* < 0.006 Change in SARA score mainly due to the reduction in its posture and gait subscore Individual goal attainment (GAS score) Improvement pre-post treatment E2/E4: *p* < 0.002 Mean score at E4: 0.45 ± 0.6 Postural sway Sitting eyes closed: unchanged Eyes open: improved (*χ*^2^ = 8.5; *p* = 0.03) These improvements correlated with postural and gait subscores of SARA score
Santos, 2017	Patients referred to the Movement Disorder Unit, Department of Neurology of a Clinical Hospital, for treatment in the Otoneurology/Rehabilitation Department, of a large private University, with a diagnosis of SCA	20 sessions of 50 min with virtual reality (VR). A Nintendo Wii hand-held remote and Wii balance board were used. Four games were played: Soccer Heading, Table tilt, Tightrope Walk, and Ski Slalom.	28 patients included. None of the enrolled patients were lost to follow-up. The DHI, BBS, and SF-36 were administered before and after rehabilitation.	1	28 (M 20; F 8) Mean age 41.6 ± 16.9 years (range 15–70) Mean disease duration 13.3 ± 12.4 20 dominant spinocerebellar ataxia, 8 autosomal-recessive ataxia	NA	DHI Pre-training Soccer heading *R* = 0.0005 *p* = 0.9978 Table tilt *R* = 0.1503 *p* = 0.4453 Tightrope *R* = 0.1310 *p* = 0.5065 Ski slalom *R* = 0.0376 *p* = 0.8495 Post-training Soccer heading *R* = 0.3589 *p* = 0.0607 Table tilt *R* = 0.5112*p* = 0.0054 Tightrope *R* = 0.4779 *p* = 0.0101 Ski slalom *R* = 0.3706 *p* = 0.0522 BBS (EEB) Pre-training Soccer heading *R* = 0.0677 *p* = 0.7322 Table tilt *R* = 0.3578 *p* = 0.0616 Tightrope *R* = 0.5771 *p* = 0.0013 Ski slalom *R* = 0.0675 *p* = 0.7327 Post-training Soccer heading *R* = 0.0929 *p* = 0.6381 Table tilt *R* = 0.4268 *p* = 0.0235 Tightrope *R* = 0.4205 *p* = 0.0259 Ski slalom *R* = 0.0542 *p* = 0.7843 SF-36** Pre-training -FC Tightrope *R* = 0.4749 *p* = 0.0107 -P Ski slalom *R* = 0.5262 *p* = 0.0040 Post-training -FC Soccer heading *R* = 0.5186 *p* = 0.0047 Table tilt *R* = 0.6429 *p* = 0.0002 Tightrope *R* = 0.5415 *p* = 0.0029 -MH Ski slalom ***R*** = 0.4220 *p* = 0.0253
Ilg, 2012	Patients recruited from the ataxia clinic of the University of Tübingen, Germany, with progressive degenerative ataxia	8-week video game–based training (available games for Microsoft Xbox Kinect) 2-week laboratory training + 6-week home training	10 patients included. Patients were examined 4 times: 2 weeks before intervention (E1), immediately before the first training Session (E2), after the 2-week laboratory training period (E3), and after the 6-week home training phase (E4)	3	10 patients (M 5; F 5) Mean age 15.5 (range 11–20) 3 autosomal-recessive ataxia, 4 Friedreich ataxia, 2 autosomal-dominant ataxia, 1 ataxia with oculomotor ataxia type 2	NA	SARA score Average score reduction: − 2 points pre/post-intervention (Wilcoxon signed-rank test: E2/E3: *p* < 0.02, E2/E4: *p* < 0.001) E1/E2: unchanged (*p* = 0.62) Reduction in SARA posture subscore: (*χ*^2^ = 5 18.4, *p* = 0.0003) Correlation with the training intensity (*r* = 20.62*, p* = 0.05) DGI DGI increase (*χ*^2^ = 8.2, *p* = 0.04; E2/E4: *p* = 0.01) ABC score 7 of 10 patients showed an increase between E2 and E4 (not statistically significant on the group level)
Assessment/diagnosis							
Arcuria 2019	Patients enrolled in the Department of Medical and Surgical Sciences and Biotechnologies (DSBMC), “Sapienza” University of Rome	Intra-rater reliability, internal consistency, and accuracy of the 15-WDACT Application for touch screen devices was measured against 9HPT and Click Test, and reliability of the app was measured over time with 4-week test-retest in 21 patients.	87 patients and 170 healthy subjects included None of the enrolled participants was lost to follow-up.	4	87 patients with ataxia (36 FA, 9 SCA1, 6 SCA2, 3 MERRF, 2 SCA3, 2 SCA8, 1 ARSACS, 1 SCAR8, MSA-C, 26 CA with no defined genetic analysis) 36M; 51F Mean age 45.6 ± 13 (range 22–76)	170 healthy subjects 85M;85F Mean age 41.36 ± 14.68 (range 18–75)	15-WDACT in healthy subjects No gender differences: *p* > 0.05 Age class differences (18–45 vs 46–75): *p* < 0.001 15-WDACT in patients Differences according to severity of symptoms (SARA score): *p* < 0.001 Correlation between increase in average execution time and severity of symptoms (*R* = 0.91) High correlation between measurements obtained with the 15-WDACT and the scores obtained with the 9HPT and Click Test Intra-rater reliability: Mean 19.91; CV 0.058 (5.8%); SD: 1.23 ICC_2.1_: 0.98 (95% CI 0.97–0.99); SEM 0.173; MDC95: 0.482 (2.4%); *α*: 0.98; *p* < 0.001
Bonnechere, 2018	Patients enrolled in the European Friedreich ataxia Consortium for Translational Studies	3 sessions with one mini-game specially developed for physical rehabilitation with spatial displacement recorded by a Kinect sensor Player had to clean the screen covered by some virtual fog using a tissue controlled by mediolateral and inferior-superior displacements of the upper limb (wrist) relative to the trunk Patients were asked to play the games three times. The mean of the three repetitions was used for statistical analysis.	27 patients and 43 healthy subjects included. None of the enrolled participants was lost to follow-up.	4	27 patients with Friedreich ataxia Mean age 26.0 (SD 12.2) Disease duration 15.0 (SD 7.44)	43 healthy subjects M 23; F 20 Mean age 26 (SD 11) years	Differences in SG between patients and healthy subjects Time (s) *t*(68) = 7.22, *p* < 0.001 Accuracy (%) *t*(68) = 3.69, *p* < 0.001 DOT (cm) *t*(68) = 2.24, *p* = 0.026 Area (cm^2^) *t*(68) = 0.74, *p* = 0.458 RMSML (cm) *t*(68) = 2.38, *p* = 0.018 RMSTD (cm) *t*(68) = 3.06, *p* = 0.003 RML (cm) *t*(68) = 2.86, *p* = 0.005 RTD (cm) *t*(68) = 2.27, *p* = 0.024 MVML (cm/s) *t*(68) = 5.23, *p* < 0.001 MVTD (cm/s) *t*(68) = 6.61, *p* < 0.001 TMV (cm/s) *t*(68) = 5.19, *p* < 0.001 Correlation between SG and genetic/clinical parameters of disease severity Correlation between disease duration and time (Pearson’s correlation coefficient= 0.64 *p* = 0.002) Correlation between disease duration and accuracy (Pearson’s correlation coefficient= − 0.67 *p* = 0.001)

*FA*, Friedreich’s ataxia; *SCA*, spinocerebellar ataxia; *CA*, cerebellar ataxia; *MERRF*, myoclonic epilepsy with ragged red fibers; *ARSACS*, autosomal-recessive spastic ataxia; *SCAR*, autosomal-recessive ataxia; *MSA-C*, multiple system atrophy–cerebellar type; *NA*, not applicable; *NR*, not reported; *DHI*, Dizziness Handicap Inventory; *BBS*, Berg Balance Scale; *SF-36*, Short-Form 36-Item; *FC*, functional capacity; *P*, pain; *MH*, mental health; *SARA*, Scale for the Assessment and Rating of Ataxia; *DGI*, Dynamic Gait Index; *ABC*, activity-specific balance confidence; *DOT*, total displacement of the wrist related to the trunk; *ML*, mediolateral; *MV*, mean velocity; *R*, range; *RMS*, dispersion of the trajectory from the mean position; *TD*, top-down; *TMV*, total mean velocity

*Total number of questions answered as “yes” when applying the JBI CPS tool

**For SF-36, only significant values of *p* have been reported in the table

The overall methodological quality of all included studies was medium-low, with 1 trial reaching a score of 4 [26], 2 trials having a score of 3, 1 trial having a score of 1 [29], and both diagnostic studies having a score of 4.

The main reason for low scores in trials was that 3 studies did not include a control group [27–29]; thus, patients were not randomized nor blinded. All trials included small samples (ranging from 9 to 28 participants), and the only randomized study [26] applied a block randomization in 9 subjects, thus leading to significant differences (e.g., Walking speed, Nine-hole peg test) in the characteristics of subjects between the experimental and the control group. Moreover, 1 trial [29] reported and analyzed data in an unclear way, leading to a high risk of incomplete and selective reporting (e.g., means and ICs are not reported). However, ataxias are relatively rare conditions, the technologies tested are difficult to standardize, and it is relatively difficult to blind participants to them.

The main risk of bias in diagnostic studies was that they were designed as case-control studies, thus the reference standard was carried out before the index test, and raters were not blind to the diagnoses. However, the technology adopted was objective; thus, the risk of interpreting results in an altered way due to the knowledge of the diagnosis is relatively low. Both trials enrolled small samples of selected patients (e.g., from a consortium), and one study enrolled subjects with different diagnoses. As previously stated, ataxias are relatively rare diseases, thus enrolling a large number of consecutive or random subjects might be very difficult.

**Fig. 2:**
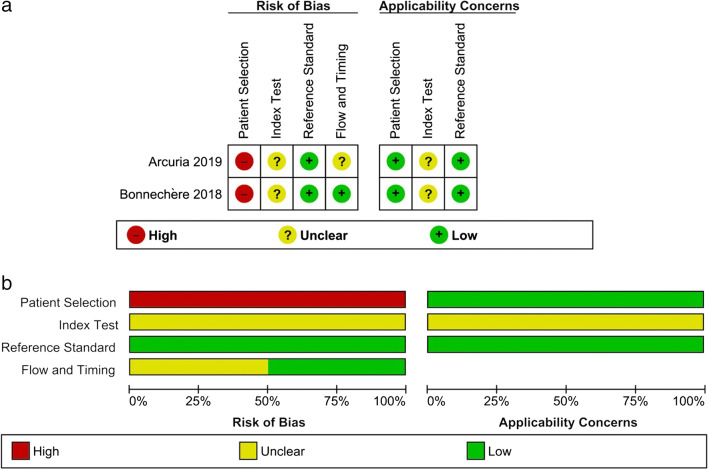
Results from the risk of bias assessment of included diagnostic studies. **a** Summary table of the assessment for each item for each study. **b** Graph plotting the distribution of assessments across studies for each item

**Fig. 3:**
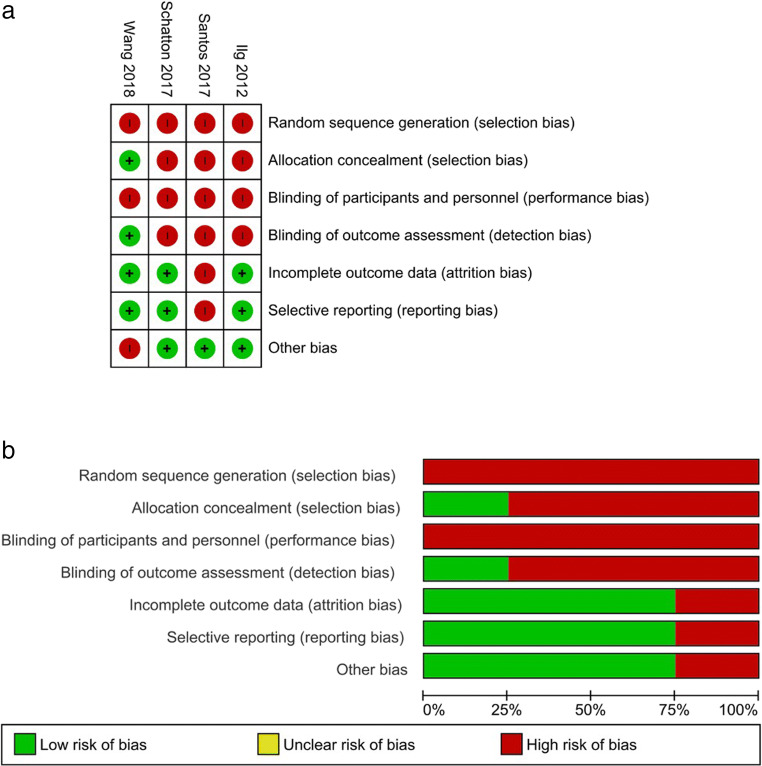
Results from the risk of bias assessment of included RCTs. **a** Summary table of the assessment for each item for each study. **b** Graph plotting the distribution of assessments across studies for each item

A summary of the qualitative assessment of all included studies is reported in Fig. 2 for diagnostic studies and Fig. 3 for trials.

As for the included trials, a high heterogeneity was observed in the characteristics of the populations enrolled, in the type of interventions used, and in the outcomes considered.

Three studies included participants aged < 18 years, while 1 included only adult subjects. Age ranges were extremely heterogeneous, with 1 study enrolling subjects aged 11–20 [28], 1 enrolling subjects aged 15 to 70 [29], 1 including subjects aged 6–29 [27], and 1 including adults aged 51–60 [26].

All 4 trials used exergaming for the rehabilitation of both adults and children with different types of ataxia, and all employed commercially available consoles and video games. Specifically, 1 study used the Nintendo Wii [29], 1 used the Xbox Kinect [28], 1 used both the Nintendo Wii and the Xbox Kinect [27], and 1 used the Kinect sensor along with a specific exergame program [26].

Only 1 study included a control group treated with conventional training [26]; all the remaining studies were based on “inter-subject” control, meaning that they compared performances pre- and post-training.

**Fig. 4:**
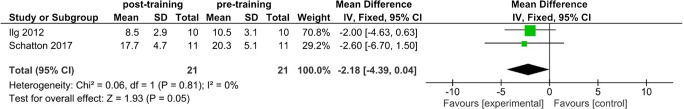
Results from the meta-analysis of the subgroup of homogeneous studies

Three trials used the SARA score as their primary outcome measure, while 1 used the Dizziness Handicap Inventory (DHI), the Berg Balance Scale (BBS), and the Short-Form 36-Items (SF-36) scores as outcome measures [29]. Considering the relative homogeneity of the outcome measure in three trials, we attempted a meta-analysis of the mean change from baseline to post-training in the SARA score. However, 1 trial [26] did not report the mean scores and SD pre- and post-training; thus, we excluded it from the meta-analysis. Means and SDs of the remaining 2 trials [27, 28] were calculated using the individual patient data reported in the studies, and for 1 of the trials, post-test scores [28] were extracted from the provided graph (Fig. 4).

### Description of Included Studies

The study by Wang et al., the only controlled study [26], was a pilot trial enrolling 9 subjects with spinocerebellar ataxia (SCA) type 3 aged 51 to 60 years (mean age 54) randomized to either exergaming with Kinect sensor or to conventional balance and coordination training. The experimental group (*n* = 5) followed 3 sessions of 40-min per week for 4 weeks of an exergaming program intervention (a specifically designed game including a reaching task, a pointing task, a following task, and an avoiding task) using a Kinect sensor. Results showed a median decrease in the SARA score of − 30.0% (range − 42.9–− 25.0) in the exergaming group and − 16.8% (− 33.3–− 8.3) in the control group, with a reduction up to − 50% of the gait-posture subscore in the exergaming group.

Among non-controlled studies, the trial by Santos et al., the largest one [29], which was also the one with the lowest quality score, enrolled 28 subjects with undefined SCA (*n* = 7), SCA3 (*n* = 5), SCA10 (*n* = 5), SCA2 (*n* = 2), SCA4 (*n* = 1), and autosomal-recessive cerebellar ataxia (ARCA) (*n* = 8). All subjects underwent a therapeutic body-balance training for vestibular rehabilitation using the Nintendo Wii hand-held remote and Wii balance board with 4 commercially available balance games (Soccer heading, Table tilt, Tightrope walk, Ski slalom). Results report an improvement in balance, measured with the Berg Balance Scale (BBS), and in dizziness, measured with the Dizziness Handicap Inventory (DHI). However, data are not clearly reported in the publication and crude values pre- and post-training for each outcome measure are not detailed.

The study by Schatton et al. [27], another non-controlled trial, enrolled 11 subjects diagnosed with Friedreich ataxia (FA) (*n* = 5), ataxia telangiectasia (*n* = 3), autosomal-recessive ataxia with no identified genetic cause (*n* = 2), or ataxia with oculomotor ataxia type 1 (*n* = 1). All participants underwent 12 weeks of coordinative training with Nintendo Wii and Xbox Kinect using commercially available video games. The study used the SARA score as the main outcome measure, and reported a significant short-term (at 6 weeks; *p* < 0.002) and long-term (at 12 weeks; *p* < 0.006) improvement in the overall SARA score from baseline to post-training, mainly driven by improvements in posture and gait.

The study by Ilg et al., the smallest of the non-controlled trials [28], enrolled 10 patients with FA (*n* = 4), ataxia with oculomotor ataxia type 1 (*n* = 1), and autosomal-recessive (*n* = 3) and autosomal-dominant (*n* = 2) ataxia without genetic diagnosis. All patients underwent an 8-week video game–based coordination training using 3 commercially available Microsoft Xbox Kinect games (Table tennis; Light race; 20,000 Leaks). The study used the SARA score as the primary outcome measure, with results showing a significant short-term (at 2 weeks; *p* < 0.02) and long-term (at 8 weeks; *p* < 0.001) improvement in the overall score from baseline to post-training, mainly due to an improvement in posture (*p* = 0.0003). Results also showed a significant improvement in the overall dynamic balance (*p* = 0.01).

As for diagnostic studies, the study by Arcuria et al., the largest one [31], enrolled 87 patients affected by FA (*n* = 36), SCA1 (*n* = 9), SCA2 (*n* = 6), myoclonic epilepsy with ragged red fibers (MERRF) (*n* = 3), SCA3 (*n* = 2), SCA8 (*n* = 2), autosomal-recessive spastic ataxia of Charlevoix-Saguenay (ARSACS) (*n* = 1), autosomal-recessive ataxia type 8 (SCAR8) (*n* = 1), multiple system atrophy-cerebellar type (MSA-C) (*n* = 1), or CA with no defined genetic diagnosis (*n* = 26), as well as 170 sex- and age-matched healthy subjects. All participants were tested with the 15-White Dots APP-Coo-Test (15-WDACT), an application specifically developed for tablets-PC, as index test, and the Nine-hole peg test (9HPT) and Click Test as reference standards. In healthy subjects, results showed no significant differences based on gender, while a significant difference in the mean execution time between the 18–45 age class and the 46–75 age class was reported (*p* < 0.001). In patients, 15-WDACT execution time increased along with disease severity (*R* = 0.91), and a high correlation was observed between measurements obtained with the 15-WDACT and the scores obtained with the 9HPT and Click Test. Moreover, results showed a high intra-rater reliability, accuracy, and internal consistency.

The study by Bonnechere et al., the smaller study classified as diagnostic [30], enrolled 27 patients with FA and 43 healthy subjects. All subjects participated in 3 sessions of a specifically designed mini-game (Wipe Out game), with spatial displacement recorded by a Kinect sensor. Highly significant differences were observed for time and accuracy of execution between patients and controls (both *p* < 0.001). Among patients, significant correlations were observed between age and time of execution (*p* = 0.015) and accuracy (*p* = 0.004), as well as between age at diagnosis and speed-related parameters (*p* = 0.021). A significant correlation was also observed between the Nine-hole peg test and the total displacement of the upper limbs (*p* = 0.012), the area covered (*p* = 0.025), the amplitudes of movements (mediolateral displacement *p* = 0.041; top-down displacement *p* = 0.033). Moreover, a statistically significant correlation was found between disease duration and decreased speed (*p* = 0.002), which was associated with a significant reduction in accuracy (*p* = 0.001).

## Discussion

In this review, we assessed currently available evidence on the use of new mobile and gaming technologies in the assessment and rehabilitation of people with chronic ataxias. Six studies were included in the systematic review, enrolling a number of participants ranging from 9 to 28 in trials and from 70 to 248 in diagnostic studies. The wide difference in the sample sizes between the 2 types of study might be due to the fact that treating a patient within a structured rehabilitative intervention is extremely more challenging in terms of resources than assessing a motor feature. Two of the included studies were diagnostic, while 4 were experimental studies. Although we found a small number of trials and despite their low methodological quality, all of them reported an improvement of motor outcomes and quality of life as measured by specific scales, including the SARA, BBS, DHI, and SF-36 scores. The main reason for such low quality in trials was that most of them were small and uncontrolled, thus non-randomized and unblinded. Ataxias are rare diseases, thus enrolling large samples can be extremely challenging. In addition, a group of patients cannot be eligible for rehabilitation protocols based on gaming and virtual or augmented reality, due to the wide range of comorbidities potentially associated with ataxia. Among these, visual loss, hearing disturbances, intellectual disability, and predominant non-ataxia movement disorders (e.g., spasticity, chorea, Parkinsonism) are usually considered exclusion criteria [27–29]. Moreover, managing a rehabilitative study on patients that, though having the same diagnosis may have widely different phenotypic variants and peculiarities, might be very difficult. However, adopting a multicenter approach and involving organizations of ataxic patients could allow enrolling a larger number of participants, increasing the size of subgroups with homogeneous phenotypes.

We found only 2 diagnostic studies investigating the use of these technologies for the assessment of specific motor functions in people with chronic ataxias. Though having an overall low-quality score, they both reported these tools to be useful and reliable. The low quality of these studies was mainly due to their being designed as case-control diagnostic studies and the enrollment of subjects with different diagnoses, disease duration, and degree of severity. The rarity of the disease, however, makes it virtually impossible to design conventional diagnostic studies.

Overall, a wide heterogeneity was observed across included studies in the participants enrolled, the type of technologies applied, and the outcome measures adopted. This prevented a direct comparison and a cumulative analysis of results. Some studies enrolled patients with different types of ataxias, different age classes, and different levels of severity of symptoms. Only 2 studies used the same technologies, with the same outcome measures. As a result, it is difficult to draw general conclusions on the most appropriate approach and the optimal age of intervention for the investigated diseases.

An additional limitation is that all included studies enrolled participants with progressive (degenerative) ataxias, mostly characterized by cerebellar atrophy and a progressively disabling course. None of the studies included patients with non-progressive forms, which are typically associated with midbrain-hindbrain malformations (mostly cerebellar hypoplasia and/or dysplasia of variable severity, either isolated or associated to other brainstem defects) [32, 33]. Considering the almost stable course of neurological impairment associated with non-progressive ataxias, it is reasonable to expect a greater and more durable effectiveness of gaming-based rehabilitation in this kind of patients. However, further targeted studies are needed to address this issue.

Video games, exergames, serious games, and apps were proven to be safe, feasible in patients with Parkinson’s disease, and at least as effective as traditional rehabilitation [34] specifically in improving balance and fatigue [35], and they also appear to be promising tools in the treatment of children with cerebral palsy [36]. Therefore, further, and more high-quality, studies should be carried out on the use of these technologies in people with different types of ataxia. The ideal study to investigate the efficacy and/or effectiveness of any type of interventions would be a randomized, controlled trial. To minimize bias, trials should include a large sample of consecutive patients and randomize them to either treatment or placebo/usual care in a blinded fashion. Maintaining the blindness of both participants and the staff administering the treatment, however, can be impossible, in rehabilitative trials; thus, usually the blindness of the personnel assessing the outcomes should be at least guaranteed. Therefore, additional larger, multicenter randomized controlled trials should be carried out with the objective of providing more structured evidence of the potential efficacy and effectiveness of exergaming in the rehabilitation of children and adults with ataxia.

## Conclusion

Results from the included studies were inconclusive, as there was a wide heterogeneity in the considered outcomes and in the type of games and technology adopted; thus, results, except for two studies, could not be aggregated nor directly compared. However, games and apps appeared to be promising in improving the motor symptoms and quality of life of patients with ataxias, or in testing specific types of symptoms. Therefore, as video games, exergames, serious games, and apps were proven to be safe, feasible, and at least as effective as traditional rehabilitation tools in patients with neurodegenerative diseases, further and more high-quality studies should be carried out on the use of these promising technologies in people with different types of ataxia.
